# Measuring student perceptions of motivation and engagement with Kahoot!: a case study in primary education in Portugal

**DOI:** 10.3389/fpsyg.2026.1881419

**Published:** 2026-07-10

**Authors:** Raquel Lopes, Ana Baptista, Sergii Tukaiev, Vaitsa Giannouli, João Alves Ferreira

**Affiliations:** 1 Piaget Research Center for Ecological Human Development, Jean Piaget Higher School of Education of Almada, Almada, Portugal; 2 Department of Special Education, School Grouping of Aveiro, Aveiro, Portugal; 3 Higher Institute of Science Education and Technology, USI Università della Svizzera Italiana, Lugano, Switzerland; 4 Kyiv National University (KNU), Taras Shevchenko National University of Kyiv, Kyiv, Ukraine; 5 Department of Psychology, Democritus University of Thrace, Didymoteicho, Greece; 6 Faculty of Medicine, Institute of Pharmacology and Experimental Therapeutics, University of Coimbra, Coimbra, Portugal; 7 Coimbra Institute for Clinical and Biomedical Research (iCBR), Faculty of Medicine, University of Coimbra, Coimbra, Portugal; 8 Center for Innovative Biomedicine and Bio-Technology (CIBB), University of Coimbra, Coimbra, Portugal

**Keywords:** formative assessment, gamification, inclusive education, motivation, student engagement

## Abstract

The integration of digital tools in teaching remains a challenge for many educators, often due to resistance to change and limited training. Drawing on Self-Determination Theory, as proposed by Deci and Ryan, and Cognitive Load Theory, developed by Sweller, this exploratory study examines student motivation and engagement in a gamified learning environment. Specifically, it explores students' perceptions of the use of Kahoot! in the classroom and the extent to which the tool may add value to the teaching and learning process. The study involved 20 fourth-grade students and their class teacher from a public school cluster in the Central region of Portugal. Data were collected through a student questionnaire, a semi-structured teacher interview, and audiovisual recording of the classroom session. Descriptive findings suggest that students perceived Kahoot! positively, particularly in terms of enjoyment, engagement, perceived usefulness, and peer interaction, while the teacher highlighted its usefulness for real-time formative assessment. However, technical constraints and connectivity problems limited implementation. Given the exploratory design, small convenience sample, dichotomous items, and absence of validated psychometric measures and a control group, the findings do not support causal inference. The study therefore contributes context-specific evidence and points to the need for future research using experimental designs and validated instruments.

## 1 Introduction

Contemporary society, marked by rapid social and technological change, requires education to prepare young people not only with solid scientific knowledge but also with the intellectual, personal, and social competencies needed to respond to changing realities (Marques and Pinto, 2014). This makes it necessary to rethink educational practices still grounded in models inherited from the Industrial Revolution, which are based on the standardization of time, space, schedules, and work sequences (Vieira, 2013). Such models tend to promote passive learning and remain closely associated with teacher-centered approaches, in which students are positioned primarily as recipients of information (Barbosa and Moura, 2014; Eison, 2010).

This standardized approach does not adequately respond to the diversity of students' rhythms, interests, and abilities, nor does it prepare them for a knowledge society that values autonomy, collaboration, personalization, and interdisciplinarity (Moran, 2005). Learning should therefore enable students to construct knowledge actively, fostering discovery and the personal appropriation of meaning (Meirieu, 1992). In this context, teacher education and the integration of digital culture into educational practice emerge as fundamental strategies. Participation, collaboration, creation, and the expansion of formal and informal learning environments are strengthened by Information and Communication Technologies (ICT) and by active learning methodologies, in which students assume a central role in the learning process (Gut et al., 2023; Marques and Pinto, 2014; Valente et al., 2017).

Active learning refers to approaches in which students actively engage in knowledge construction through inquiry, problem-solving, collaboration, and reflection (Barbosa and Moura, 2014; Bonwell and Eison, 1991; García, 2024; Keiler, 2018; Prince, 2004; Weltman, 2007). Within this framework, the teacher assumes a facilitative role, guiding activities and promoting feedback rather than transmitting information directly. These activities provide opportunities for students to apply and demonstrate knowledge while receiving immediate feedback from peers and/or the teacher (Nasser Alkathiri et al., 2024; García, 2024; Fredriksen et al., 2025; Keiler, 2018; Souza et al., 2014; Park et al., 2021). They may occur inside or outside the classroom, individually, in pairs, or in groups, and may be supported by digital technologies or implemented without them (Bingen et al., 2023; Eison, 2010; Felder and Brent, 2009; Mand et al., 2024).

When supported by digital technologies, active learning may make knowledge more meaningful because it emerges from learners' engagement with the concepts under study (Berbel, 2011; Mand et al., 2024; Moran, 2005). It may also strengthen autonomy, self-confidence, decision-making, the practical application of knowledge, oral and written expression, and enjoyment in problem-solving. In addition, such practices promote social interaction, collaborative competence, and the inclusion of all members of the class as part of a shared learning community (Souza et al., 2014).

Among the most widely used active learning methodologies are Think-Pair-Share, cooperative and collaborative learning, formative assessment, and project-based work, all of which converge toward the shared goal of creating active and meaningful learning environments (Barbosa and Moura, 2014). Active learning therefore represents not merely a methodological shift, but a broader educational paradigm associated with student engagement, motivation, and academic success (Nasser Alkathiri et al., 2024; Fredriksen et al., 2025; Michael, 2006; Souza et al., 2014).

To move beyond descriptive accounts of pedagogical practice, this study grounds its analysis in established psychological theories of learning and motivation, namely Self-Determination Theory and Cognitive Load Theory. These frameworks provide a useful lens for interpreting how gamified classroom activities may influence student experience, without implying causal effects beyond the scope of the present exploratory design.

### 1.1 Theoretical framework: psychological mechanisms of motivation and engagement

To interpret students' experiences in a theoretically informed manner, the present study draws on two complementary perspectives: Self-Determination Theory (SDT) and Cognitive Load Theory (CLT). Together, these frameworks make it possible to examine not only whether students respond positively to a gamified activity, but also which motivational and cognitive processes may help explain these responses.

#### 1.1.1 Self-determination theory

Self-Determination Theory (SDT; Deci and Ryan, 1985; Ryan and Deci, 2020) proposes that intrinsic motivation, which is associated with deeper learning, persistence, and wellbeing, emerges from the satisfaction of three basic psychological needs: autonomy, competence, and relatedness. In classroom settings, gamified tools such as Kahoot! may potentially support these needs through immediate performance feedback, collaborative interaction, and structured opportunities for participation. At the same time, competitive elements may also threaten autonomy and relatedness if they are not carefully calibrated to the characteristics of the learners and the learning environment (Reeve and Cheon, 2021).

#### 1.1.2 Cognitive load theory

Cognitive Load Theory (CLT; Sweller, 1988; Paas and Sweller, 2012) offers a complementary lens. CLT distinguishes intrinsic cognitive load, inherent to task complexity, from extraneous load, imposed by instructional design, and germane load, directed toward schema construction. Gamification elements such as timers, sound effects, and leaderboards may increase extraneous load and potentially interfere with learning (Paas and Sweller, 2012). Conversely, immediate feedback and spaced repetition may reduce intrinsic load by facilitating automatization of knowledge (van Merriënboer and Sweller, 2005). This tension between gamification as a facilitator and as a possible disruptor remains insufficiently explored in primary education contexts.

#### 1.1.3 Student engagement as outcome

Student engagement, conceptualized as a multidimensional construct encompassing behavioral, emotional, and cognitive components (Fredricks et al., 2004), serves as the integrative outcome variable. Rather than treating engagement as a simple indicator of enjoyment, the present study considers it as a broader construct shaped by motivational, social, and cognitive processes. In this sense, the structural features of Kahoot! are examined as possible triggers of such processes, generating theoretically informed propositions rather than causal claims.

### 1.2 Active learning environments supported by ICT in the Portuguese context

Despite progress achieved within the Portuguese educational system, there remains a need to rethink traditional teaching methodologies and to promote student-centered processes, critical thinking, and active participation (Alves, 2017). Recent educational policies have emphasized the integration of ICT as a driver of innovation, inclusion, and curriculum flexibility (Decree-Law No., 54/2018, 55/2018). Despite this progress, challenges remain, including limited teacher training and institutional constraints, which continue to hinder the effective pedagogical use of digital tools (Cohen and Fradique, 2018; Gómez, 2015).

Digital technologies are now understood not merely as supporting resources but as catalysts for new ways of constructing knowledge, enabling greater personalization, equity, and student engagement (National Education Council, 2023; Madrid, 2006). In this context, methodologies incorporating gamification have been identified as effective strategies for making learning more motivating and participatory (Almeida, 2018; Dellos, 2015).

However, the adoption of ICT continues to face barriers such as low levels of digital literacy among teachers, institutional constraints, and insufficient pedagogical training, all of which hinder innovation (Alves, 2017; Azevedo and da, 2023; Fernández-Batanero et al., 2021; Koch and Fehlmann, 2024). Teacher training for the integration of active methodologies and digital resources is therefore imperative (Bacich et al., 2015). European innovation projects such as iTEC, Creative Classroom Lab, and Future Classroom Lab have outlined pedagogical scenarios that combine active learning, digital integration, and curricular innovation.

### 1.3 Gamification and Kahoot!

Within this context, gamification has emerged as a strategy to make learning more engaging and participatory (Correia et al., 2009; Dellos, 2015; Triantafyllou et al., 2025). Gamification refers to the application of game elements and principles in non-game contexts (Deterding et al., 2011) and it has been associated with learning across cognitive, emotional, and social domains through the use of rules, challenges, problem-solving, curiosity, joy, persistence, cooperation, identity, and recognition (Deterding et al., 2011; Huizinga, 2012; Lee and Hammer, 2011; Vygotsky, 1991). More than relying on extrinsic motivation through rewards, gamification seeks to foster intrinsic motivation by developing self-determination, curiosity, engagement, and a sense of competence (Alves, 2017; Bai et al., 2020; Mendes et al., 2025).

In this process, the teacher acts as a mediator, carefully selecting ludic elements and aligning them with learning objectives (Grando, 2000; Huang and Soman, 2013). Despite its potential, barriers to implementation persist, including the perception among some educators that gamification is merely entertainment, the lack of specific training, and associated costs. Nevertheless, when appropriately integrated, gamification can reinforce motivation, critical thinking, and collaborative learning (McGonigal, 2011; Silva, 2019).

#### 1.3.1 Kahoot! as a gamification tool

Among gamified digital tools, Kahoot! has gained particular prominence due to its simplicity, accessibility, and capacity to combine real-time assessment with game-based elements (Özdemir, 2025; Toscano, 2018).

According to Wang and Tahir (2020), Kahoot! temporarily transforms the classroom into a game show, in which the teacher acts as host and students compete in real time by responding quickly and accurately on their digital devices. The most common format is the quiz, consisting of multiple-choice questions with a timer and variable scoring. The system provides immediate feedback, performance charts, and exportable reports, making it a valuable tool for formative assessment (Costa and Oliveira, 2015; Toscano, 2018).

New features—such as the answer streak bonus, team mode, and ghost mode—expand the pedagogical potential of the platform by stimulating motivation, quick thinking, and collaboration (Guimarães, 2015; Wang and Tahir, 2020). The application also allows students to create their own quizzes, promoting engagement, agency, and collaborative work, as well as the integration of multimedia elements that may support inclusion for students with visual or auditory impairments. Kahoot! therefore brings together three central dimensions—gamification, interaction, and real-time assessment—and establishes itself as a tool capable of energizing learning and fostering motivation, collaboration, and educational success (Costa and Oliveira, 2015; Guimarães, 2015; Toscano, 2018; Triantafyllou and Georgiadis, 2022; Wang and Tahir, 2020).

#### 1.3.2 Objectives and research questions

Against this background, the present study aims to provide a context-bound exploratory account of how Kahoot! is experienced in a specific primary classroom. More specifically, it seeks to examine whether and how the structural features of Kahoot! may be interpreted, through the lenses of SDT and CLT, as supporting motivational and engagement processes in primary education. Rather than testing causal hypotheses, which would require experimental designs, control groups, and validated instruments, the study seeks to connect field observations with psychological theory in order to generate theoretically grounded propositions for future research.

The central objective was therefore defined as analyzing students' perceptions of the introduction of Kahoot! as an innovative pedagogical tool in the classroom. To operationalize this objective, several dimensions of the teaching-learning process were considered, namely motivation, engagement, effort, interest, attention, social interaction, competition, and collaborative work among students.

The following research questions were formulated: (i) How do students perceive the introduction of Kahoot! in terms of autonomy, competence, and relatedness? and (ii) to what extent does the design of Kahoot! influence cognitive engagement and extraneous cognitive load in the classroom? Given the exploratory nature of the study and the absence of validated psychometric instruments, the constructs derived from Self-Determination Theory and Cognitive Load Theory are not measured directly, but are instead used as interpretative lenses to structure the analysis of observed patterns.

## 2 Materials and methods

### 2.1 Sample

This case study involved 20 children aged between 8 and 10 years, of both sexes, attending the fourth year of Primary Education in a cluster of primary schools in the municipality of Aveiro (Table 1). The sample included 7 boys and 13 girls and was selected intentionally through convenience sampling, that is, as a non-probabilistic sample (Pardal and Correia, 1995).

**Table 1:** Sample characteristics of the fourth-year children from the primary education school cluster in the municipality of Aveiro.

Age (years)	8	9	10
Male (*n* = 7)	1	5	1
Female (*n* = 13)	2	11	0
Total (*n* = 20)	3	16	1

No student had previously participated in programes related to the topic under study, and no student refused to participate. Informed consent was obtained from the students' legal guardians for the use of the collected responses. Audiovisual data were securely stored, anonymised, accessed only by the research team, and scheduled for deletion after the completion of the study.

Because the sample was selected by convenience, the findings are not statistically generalizable. They should therefore be interpreted as context-specific observations intended to generate theoretical propositions rather than evidence of population-level effects.

### 2.2 Study design

The study followed a mixed-methods design, combining quantitative and qualitative data collection and analysis. In addition to a critical review of the literature on digital tools in educational contexts, a case study was conducted on the use of Kahoot! in the classroom, specifically in quiz mode.

### 2.3 Data collection instruments

Three data collection instruments and techniques were used:

(i) A researcher-administered questionnaire applied to students immediately after the pedagogical session, consisting mainly of closed-ended yes/no questions and four open-ended questions. The items were designed to be clear, simple, and age-appropriate (Quivy and Campenhoudt, 2005). The questionnaire items were not designed to operationalize SDT or CLT constructs through validated scales; therefore, any interpretation related to autonomy, competence, relatedness, or cognitive load should be understood as indirect and exploratory.
(ii) An exploratory interview with the class teacher (*n* = 1), focused on the design and implementation of the activity, as well as perceptions regarding the use of Kahoot!. The interview guide included questions on professional background, experience with ICT training and digital tools, and open-ended questions on the perceived value of the platform.
(iii) Audiovisual recording of the classroom session, used to complement the data on student participation and interaction.

To enhance transparency and clarify the relationship between research questions, theoretical constructs, and data collection methods, Table 2 presents the alignment between these elements.

**Table 2:** Alignment between research questions, constructs, instruments, and data sources.

Research question	Construct	Instrument	Type of data
RQ1: Students' perceptions of autonomy, competence, and relatedness	Autonomy, competence, relatedness (SDT)	Student questionnaire	Indirect self-report
RQ2: Cognitive engagement and extraneous cognitive load	Cognitive engagement/extraneous cognitive load (CLT)	Observation and student questionnaire	Interpretative

### 2.4 Methodological limitations

The student questionnaire was developed *ad hoc* for this study and consisted mainly of dichotomous items. This choice limited measurement sensitivity and prevented inferential statistical analysis. The absence of validated instruments, such as the Situational Motivation Scale (SIMS; Guay et al., 2000), the Academic Motivation Scale (AMS; Vallerand et al., 1992), or the NASA Task Load Index (NASA-TLX; Hart and Staveland, 1988) for cognitive load, constitutes an important methodological limitation and restricts the psychometric robustness of inferences regarding motivation and cognitive processing. Consequently, the quantitative results are presented as descriptive trends only.

The interview transcription and coding were conducted by a single researcher. Although categorization followed principles of mutual exclusivity and homogeneity (Bardin, 1995), the absence of independent coding by multiple raters and the impossibility of calculating inter-coder reliability represent limitations that reduce coding objectivity.

### 2.5 Intervention phases: application of the pedagogical tool—Kahoot!

The intervention took place in Mathematics, using Kahoot! as an alternative to a traditional assessment worksheet. The intervention took place in Mathematics, using Kahoot! as an alternative to a traditional assessment worksheet (Table 3). The procedure was divided into two stages:

(i) A familiarization phase, in which a preliminary quiz with general knowledge questions was used to introduce students to the tool and allow initial contact with its dynamics;
(ii) A pedagogical application phase, in which a second quiz containing 20 questions related to the Mathematics curriculum was administered.

**Table 3:** Final student feedback.

Feedback	%
Enjoyment rating	Mean = 4.3/5
Learned something	15/20 (75%)—Yes
	5/20 (25%) - No
Would recommend Kahoot!	17/20 (85%) - Yes
	3/20 (15%) - No
Emotional response	17/20 (85%) - Positive responses
	3/20 (15%) - Negative responses

Percentages were calculated from *n* = 20.

The class was organized into 10 pairs of students according to device availability. To preserve anonymity, the groups participated using standardized nicknames, from “student1” to “student10.”

The game followed the typical Kahoot! structure: the question and response time were displayed, students selected their answers on their laptops, the correct answer and class statistics were shown immediately, and the scoreboard was updated on the classroom interactive whiteboard. The activity ended with the final podium display and the collection of students' feedback on the gamified experience through Kahoot! itself.

### 2.6 Data processing and analysis

Given the dichotomous nature of the questionnaire responses and the small sample size (*n* = 20), inferential statistical tests were neither feasible nor appropriate. Closed-ended responses were analyzed descriptively using frequencies and percentages in Microsoft Excel. The absence of parametric or non-parametric inferential procedures precludes tests of statistical significance and effect size estimation, which should be recognized as a limitation.

The open-ended responses were examined using interpretive content analysis, with recurring themes and patterns of opinion identified in the students' statements. The audiovisual records were analyzed in a complementary way, alongside the questionnaire and interview data, to identify indicators of student involvement, interaction, and motivation during the activity.

The teacher interview was organized into three parts:

(i) The first part collected professional background information through direct closed-ended questions (e.g., “Please indicate your professional qualifications”);
(ii) The second part included yes/no questions, some dependent on previous answers (e.g., “Have you attended ICT training sessions?;” “If yes, to what extent has this changed your pedagogical practice?”), in order to assess the teacher's training and pedagogical use of ICT and digital tools; (iii) the third part contained open-ended questions designed to elicit the teacher's views on the use of Kahoot! as a pedagogical tool in the classroom (e.g., “In your opinion, what are the potential benefits of using Kahoot! in the classroom?”).

The interview was fully transcribed, and recording units were identified as complete, meaningful sentences. Key ideas were then synthesized and categorized using a combination of open and closed coding, drawing on Bardin (1995). The categorization process followed the principles of mutual exclusivity, homogeneity, relevance, accuracy, and productivity.

## 3 Results

The results are presented in two parts: (i) Students' questionnaire responses and (ii) findings from the teacher interview. In line with the exploratory design of the study, the results are reported descriptively and no causal inference is made.

### 3.1 Students' responses to the questionnaire

**Figure 1:**
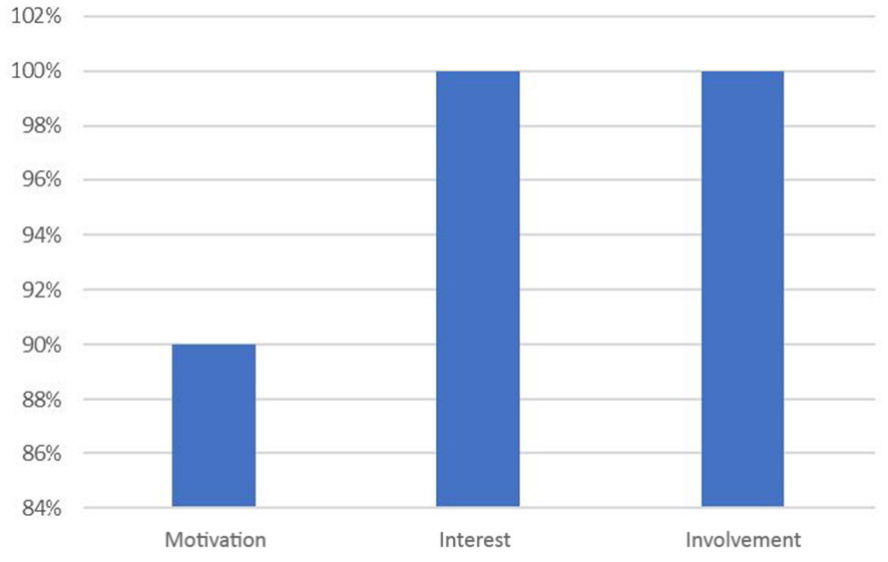
Levels of student motivation, interest, and engagement. Percentages represent the proportion of students responding positively to each item (*n* = 20).

All students (20/20, 100%) reported enjoying the use of Kahoot! in class and described it as accessible and easy to use. Students also reported high levels of motivation, interest, and engagement (Figure 1), and all indicated that they made an effort during the activity. Given the small sample size (*n* = 20), both absolute frequencies and percentages are reported.

Most students (15/20, 75%) considered Kahoot! useful for learning, and all students (20/20, 100%) indicated that it was helpful for content revision. Students also reported that the activity promoted peer interaction and collaboration, particularly because they worked in pairs to share devices and answer the questions together.

**Figure 2:**
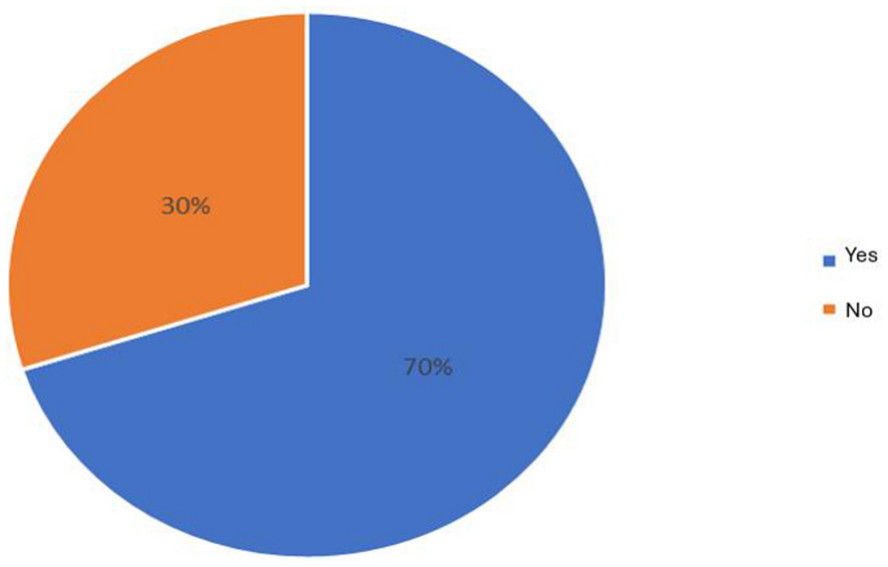
Students' views on anonymity in Kahoot!.

Most students reported valuing anonymous participation (Figure 2), stating that it reduced embarrassment and increased their sense of safety during the activity. By contrast, 4 of the 20 students (20%) preferred to have their names displayed, mainly because they associated name visibility with individual recognition on the leaderboard.

Sixteen of the 20 students (80%) reported that they appreciated the competitive element of the activity, associating it with greater effort and attention. However, 4 students (20%) reported some discomfort, mainly when they felt unprepared or when they lost. Although competition was viewed positively by most participants, it did not appear to be equally motivating for all students.

All students reported that the classroom atmosphere changed during the Kahoot! session, describing it as more playful, dynamic, and engaging. Eighteen students (90%) stated that the lesson was more enjoyable than a traditional lesson. Overall, the session received high ratings for enjoyment, and 17 students (85%) reported positive feelings after the activity.

Two students (10%) reported technical difficulties related to internet connectivity, which temporarily disrupted participation and caused frustration.

### 3.2 Teacher interview

The teacher, who had more than 20 years of professional experience, reported that she had not received initial training in ICT, although she had later attended continuing professional development courses in this area. She considered ongoing training to be very important, particularly in response to students' increasing familiarity with technology.

Regarding the preparation of the Kahoot! quizzes, she acknowledged initial difficulties, describing the process as “tedious and time-consuming,” but valued the support from colleagues. She chose not to include images or videos to avoid distractions, considering that the preparation phase was fundamental to the activity's success.

Concerning the implementation of Kahoot! in the classroom, the teacher emphasized the students' positive reception and collaborative behavior. She reported that students became more excited, attentive, and engaged, appeared closer to their peers, and showed greater commitment to completing the tasks. She also observed an improvement in classroom behavior.

The teacher stated that a familiarization phase prior to the Mathematics activity was necessary in order to improve the implementation of the tool. She also explained that students had to be organized into work groups because of equipment limitations and that anonymity was adopted to avoid embarrassment. According to her, students became more attentive, engaged, and cooperative, while her own role became less directive and students displayed greater autonomy in the learning process. She described the classroom environment as lighter, more enjoyable, interactive, and innovative, while also noting that the activity created a more competitive, although still healthy, atmosphere. The teacher's observations provide contextual triangulation but represent a single informant, limiting reliability.

When asked about the potential benefits of using Kahoot! in the classroom, the teacher highlighted several advantages, including the rapid assessment of students' understanding of previously taught content, support for the review and consolidation of learning, promotion of collaborative work, access to detailed participation reports for formative and summative assessment, and reduced paper use and marking time. She also emphasized the positive effects of the activity on student motivation, behavior, and interaction.

Observing the students during the quiz, the teacher reported that they responded in an organized manner and were aware of the time constraints. She emphasized that responses were discussed collaboratively within groups, fostering cooperation and respect for others' opinions. She also considered that students were actively involved in the teaching and learning process throughout the lesson.

When asked about constraints affecting the implementation of Kahoot! in the classroom, the teacher identified the main limitations as the insufficient number of laptops, outdated computer equipment, and internet failures that blocked some devices and temporarily discouraged some student groups. She noted that some students became visibly upset because they were unable to answer the questions. Despite these challenges, she stated that she intended to continue using Kahoot! and other digital tools.

Finally, the teacher regarded the experience as a clear departure from traditional teaching paradigms and viewed digital tools as catalysts for more dynamic, participatory, and collaborative learning. She also reported that, in primary education, most colleagues mainly used the interactive whiteboard and textbooks, with only occasional use of digital platforms for videos or exercises. In her view, the pedagogical use of digital tools in classroom practice remains insufficiently discussed, partly because of limited teacher training.

## 4 Discussion

This exploratory study examined how the structural features of Kahoot! may be associated with psychological processes related to motivation and engagement, drawing on Self-Determination Theory and Cognitive Load Theory. The findings suggest patterns broadly consistent with the satisfaction of basic psychological needs, particularly competence through immediate feedback and relatedness through collaborative participation. However, these observations should be interpreted as context-specific and exploratory rather than as evidence of effectiveness.

### 4.1 Competence and relatedness through collaborative structure

The organization of students into pairs appeared to encourage joint problem-solving and was broadly consistent with the relatedness dimension proposed by Self-Determination Theory. However, pairing emerged primarily from equipment constraints rather than from an intentionally designed collaborative strategy, an important distinction when interpreting the pedagogical meaning of the activity. The observation that students responded collaboratively and respected one another's opinions is consistent with behavioral engagement, although cognitive engagement, such as elaborative processing or metacognitive regulation, was not directly assessed.

### 4.2 Playfulness, cognitive load, and engagement

The playful nature of Kahoot!, particularly its real-time leaderboard and game-show dynamics, appeared to support emotional engagement and a more positive classroom climate. At the same time, enjoyment should not be treated as equivalent to cognitive engagement or improved learning outcomes as findings about learning across the lifespan highlight in different cultural contexts (Demetriadis et al., 2015). From a Cognitive Load Theory perspective, the platform may have increased extraneous cognitive load through time pressure and competitive pressure, while also potentially supporting learning through immediate feedback.

The teacher's emphasis on real-time formative assessment is also relevant here, as externalized feedback may reduce intrinsic cognitive load by freeing working memory for schema construction (Stoyanova and Giannouli, 2022). However, this interpretation remains speculative in the absence of objective cognitive load measures. Overall, the tension between gamification as a cognitive facilitator and as a possible source of distraction cannot be resolved on the basis of the present descriptive data, but it does generate useful hypotheses for future research.

These findings are broadly consistent with previous literature suggesting that digital technologies can enhance student motivation and participation. Almeida (2018) reported that many teachers perceived digital resources as increasing students' readiness to learn, while Nasser Alkathiri et al. (2024) showed that the active integration of digital tools may foster more meaningful learning and participation. In relation to Kahoot!, Wang and Tahir (2020) argued that its use may increase motivation, concentration, and enthusiasm. The present results align with that literature insofar as students described the activity as enjoyable and useful for revising previously taught content.

### 4.3 Active methodologies and classroom change

The findings also suggest that gamification contributed to a more active, interactive, and appealing classroom environment in which students assumed more visible and participatory roles, in line with the literature on active methodologies. This shift, also described by the teacher, is consistent with a move away from transmission-centered instruction toward a more facilitative teaching model. In this sense, Kahoot! appears to have functioned not only as a digital resource, but also as a catalyst for interaction and classroom participation.

Nevertheless, the teacher's perspective should be interpreted cautiously, as it may have been shaped by expectancy effects, novelty effects, and social desirability bias. Because the teacher was directly involved in the implementation of the activity, her perceptions may also have influenced both classroom dynamics and student responses.

### 4.4 Methodological limitations

The study has several important limitations. First, the convenience sample of 20 students from a single class, together with one participating teacher, does not allow statistical generalization. The findings should therefore be interpreted as exploratory and context-specific rather than as evidence of population-level effects. Second, the absence of a control group, pre-test measures, and randomization prevents causal inference; the observed effects cannot be attributed solely to Kahoot!, nor can they be disentangled from novelty effects, teacher enthusiasm, or classroom context.

Third, the use of dichotomous items without validated scales limits construct validity and measurement sensitivity. Claims about motivation and engagement therefore rely on self-reported indicators rather than on psychometrically robust instruments. Fourth, the qualitative coding was conducted by a single researcher, which limits objectivity and prevents the estimation of inter-coder reliability. Finally, the immediate post-intervention design does not permit any assessment of sustained effects and may increase the influence of novelty bias and socially desirable responding.

These limitations are not merely technical; they reflect the distinction between exploratory, theory-generating research and confirmatory, hypothesis-testing research. The present study clearly belongs to the former category. Even so, the findings suggest that the activity may have contributed to a more active, motivated, and collaborative learning environment. The teacher also highlighted the value of Kahoot! for formative assessment, as it enabled real-time monitoring of student responses and the generation of individualized reports, in line with Colombo and Bica (2020). Such features may support the identification of learning difficulties and encourage reflection on performance.

### 4.5 Implications and future work

In relation to the study's central objective, Kahoot! appears to represent a potentially useful pedagogical resource for promoting motivation, interest, and engagement in the classroom. It may also support collaborative work, improve classroom dynamics, and provide the teacher with more immediate evaluative information. More broadly, the findings suggest that gamification through tools such as Kahoot! may contribute to a shift away from transmissive teaching toward interaction, collaboration, and active learning.

Future research should adopt experimental designs, ideally with pre-test/post-test measures and randomized control groups, in order to test the propositions generated by this study more rigorously. The inclusion of validated instruments, such as SIMS for motivation, NASA-TLX for cognitive load, and standardized achievement measures, would allow stronger inferences and more robust mediation analyses. It would also be useful to examine moderation by individual differences, such as trait anxiety or achievement goal orientation, in order to better explain variation in responses to competition. Only through more rigorous designs will it be possible to determine whether gamification is a genuinely effective instructional strategy or merely a transient novelty effect.

## 5 Conclusions

This exploratory case study examined how the use of Kahoot! in primary education may be associated with motivational and cognitive processes interpreted through the lenses of Self-Determination Theory and Cognitive Load Theory. The findings suggest that Kahoot!'s classroom use was associated with patterns broadly consistent with competence, relatedness, enjoyment, and participation, although responses to competition were not uniformly positive.

However, the exploratory design, small convenience sample, absence of a control group, and use of non-validated instruments prevent causal inference and limit the transferability of the findings beyond the immediate study context. Rather than providing confirmatory evidence, the study offers a theoretically informed basis for understanding how gamified tools may shape students' classroom experience in primary education.

The main contribution of the study lies in the generation of theoretically informed propositions about how gamified tools may engage motivational, social, and cognitive processes in primary classroom settings. Future research should therefore adopt more rigorous designs, including validated measures, comparison groups, and longitudinal or experimental approaches, in order to determine more clearly whether the benefits associated with gamification reflect sustained pedagogical value or short-term novelty effects.

## Data Availability

The raw data supporting the conclusions of this article will be made available by the author, without undue reservation.
